# Treatment of Diarrhœa and Dysentery

**Published:** 1864-02

**Authors:** 


TREATMENT OF DIARRHŒA AND DYSENTERY—By Prof. Skoda.

Beyond everything stands a strict regulation of the diet. When the intestinal canal is in a diseased state almost any subject introduced into the stomach acts mischievously, and it is not unfrequently necessary to suspend all food until the intestine is in a condition to bear it. Every solid article *eo ipso* is then mischievous, but even fluids, by reason of their temperature, may act as prejudicially. In most cases taking a few spoonfuls of warm soup, or drinking a mouthful of cold water will immediately be followed by severe colics, and soon afterwards by evacuations. We must only allow lukewarm soups or other drinks, and that only by a spoonful at a time. Of course, these stringent rules only apply to a very obstinate diarrhœa, and especially dysentery, for there are many cases of temporary diarrhœa in which the patients continue to eat fruits and the like, and still soon get well. Such cases must, however, not be taken into account, and it is always most prudent at the commencement of a diarrhœa to cut off the supply of food as far as possible, and at all events to prohibit all articles likely to augment the affection.

Opium is the most valuable medicine in diarrhœa, for it keeps the sphincter in a state of permanent contraction, a contraction which is often propagated to the large intestine, and the small intestine is unable to propel its contents far enough to induce the irritation which causes their expulsion. When, by reason of this contraction, these contents are retained, their amount may become considerably diminished by the absorption of the fluid. Frequently, however, there is no spot of the canal which is not so diseased as to prevent such absorption taking place, and then the diarrhœa will continue in spite of the opium and of the contraction of the sphincters. It appears, moreover, that opium, besides its action on the muscular portion of the canal, exerts, by contact, a soothing effect upon the mucous membrane. In consequence of the diminution of the irritation of this membrane, its secretion is probably lessened, as are possibly those of the liver and pancreas. However this may be, opium acts very favorably in profuse secretion from the intestinal mucous membrane. From half a grain to three grains may be given in the twenty-four hours, the best preparation being the *ext. opii aquosum.*

If opium or morphia do not suffice, it must be aided by astringent remedies, by far the best of which, and the most easily supported, is the sulphas zinci. One would have supposed that tanin in its separate state would have proved more useful than the zinc, but this is not the case, and it is much less easily borne. It acts much better and more energetically when employed as a household remedy (*e. g.*, as a decoction of sloe or wild pear tree) than in its separated form; and is then of great service in practice among the poor. Alum is of no use whatever in diarrhœa. Lead approaches zinc in efficacy, but still it is less certain than it. The dose should not be greater than a quarter of a grain, and this may be repeated every two or three hours, and at most every hour. If these means do not suffice, we must have recourse to enemata of salep or starch (with which may be combined one grain of opium or half a grain of zinc) not throwing up more than two ounces at a time. If the clyster does not cause pain in the rectum, and the disease continues obstinate, the dose of the zinc may be increased to two grains. Tannin may be added to the enema, but the zinc is far more serviceable. In the most obstinate cases we must have recourse to cauterization; but this is only the case when there is a diseased condition of the lower part of the rectum. Very obstinate cases of blennorrhœa confined to the anus may be completely cured by the application of nitrate of silver in substance as high as it can be passed. The injection of a strong solution of this substance does not usually attain the same end.—*Med. Times and Gaz.*, *Sept.* 12, 1863, *from Wien Allgem. Med. Zeit. No.* 43.—*Am. Medical Journal.*

